# A Survey of Retinal Remodeling

**DOI:** 10.3389/fncel.2015.00494

**Published:** 2015-12-23

**Authors:** Enrica Strettoi

**Affiliations:** Italian National Research Council, Neuroscience InstitutePisa, Italy

**Keywords:** retinitis pigmentosa, bipolar cell, ganglion cell, mGluR6, retinal glia

## Abstract

Up to 15 years ago, bibliographic searches based on keywords such as “photoreceptor degeneration, inner retina” or “photoreceptor degeneration, second order neurons” returned only a handful of papers, as the field was dominated by the general assumption that retinal degeneration had direct effects on the sole populations of rods and cones. Since then, a number of studies have been dedicated to understanding the process of gradual morphological, molecular, and functional changes arising among cells located in the inner retina (comprising neurons, glia, and blood vessels), that is to say “beyond” photoreceptors. General aspects of this progression of biological rearrangements, now referred to as “remodeling”, were revealed and demonstrated to accompany consistently photoreceptor loss, independently from the underlying cause of degeneration. Recurrent features of remodeling are summarized here, to provide a general frame for to the various analytical descriptions and reviews contributed by the articles in the issue (*among others, see Euler and Schubert, 2015; Soto and Kerschensteiner, 2015, this issue*).

By definition, the term **remodeling** refers to the reorganization or renovation of a pre-existing structure. In visual system studies, this term has been traditionally used to describe dendritic and axonal rearrangements (i.e., dendritic pruning, projection refinement) occurring during physiological development, or to indicate vascular alterations in ocular pathologies. The concept of remodeling entered consistently the field of retinal research in 2003, when Robert Marc used this word to indicate the gradual and articulated process of morpho-functional changes involving neurons, glia and blood vessels that in the retina accompany and follow photoreceptor degeneration (Marc et al., 2003).

In previous years, most of the literature published under the general theme of “inherited retinal degeneration” was dedicated to the genetics of this complex family of disorders, to the clinical aspects of the human diseases and the kinetics of photoreceptor degeneration, with an emphasis on genotype–phenotype correlations; gene therapy was at its beginning (Berson, 1996; Farber and Danciger, 1997; Chong and Bird, 1999; Bennett and Maguire, 2000). Animal models were relatively few and the research focussed understandably on photoreceptors, whose loss is responsible for blindness, the inevitable outcome of the disease. The general idea was that photoreceptors were the only cells undergoing degeneration: indeed, the two main manifestations of retinitis pigmentosa (RP) and related disorders are apparently the process of primary death of rods and the secondary degeneration of cones (Hartong et al., 2006). Because humans are largely diurnal, cone loss and decrement of visual acuity are undoubtedly the most relevant clinical signs. Before the advent of sensitive OCT methods, the thickness of inner retinal layers was observed to decreases sensibly only at late stages of RP.

The incidence of the disease (estimated in 1:4,000) is expected to rise due to the genetic origin, the fact that RP does not interfere directly with life length and the general increase in life expectancy. Thus, it is not surprising that pathological specimens available from eye banks to study the histology of diseased retinas come usually from old donors at advanced pathological stages (Milam et al., 1998). Scarce availability of histological samples and variability of phenotype severity also contributed to the early assumption that the inner retina is only moderately affected by photoreceptor loss. Conversely, that severe remodeling of retinal layers located beyond photoreceptors accompanies the end stages of the disease is a long known concept: ophthalmology manuals refer to these phases as characterized by blood vessel attenuation, pallor of the optic disc and gliosis.

Clearly, what is relevant therapeutically is the occurrence and biological nature of *early* processes of remodeling, involving neuronal classes (i.e., bipolar and ganglion cells) critical to accurate transmission of visual information to the brain. Preservation of these neurons is relevant for they are ideally exploitable as platforms for vision restoration.

And yet, up to 15 years ago, bibliographic searches based on keywords such as “photoreceptor degeneration, inner retina” or “photoreceptor degeneration, second order neurons” returned few papers, mostly focusing on ganglion cell damage in glaucoma or describing Muller cell hyper reactivity accompanying photoreceptor loss. Few articles reported data concerning the effects on cells other than photoreceptors in RP and related disorders.

The field has grown considerably thereafter. Albeit still concentrated on photoreceptors and the implementation of rescue strategies for these cells to prolong vision (Thompson et al., 2015), the research arena of retinal degenerations has developed a culture of remodeling, with several laboratories studying this topic worldwide. It is now clear that a chain of events, mostly of regressive nature, accompany and follow photoreceptor loss leading to the gradual deconstruction of the inner retina (Jones et al., 2005). These events are important for the choice of vision restoration strategies relying on cells located beyond photoreceptors, as severe remodeling might threatened the outcome of these approaches.

Some general features of remodeling are now known: in RP, this is a process that builds up in time and arises secondarily; it is gradual, both in a temporal domain, as well as in space, taking place earlier and more severely among cells directly connected to photoreceptors (i.e., bipolar cells) and becoming milder moving toward the deepest retinal layers; finally, remodeling is quite stereotyped. Despite tremendous genetic heterogeneity of RP and allied disorders, regressive events occurring in the inner retina fall within a limited range and therefore are somehow predictable. Stereotypy is both mutation- and species- independent: although most of what is known has been obtained from animal models, the findings in rodents and rabbits are similar and parallel the limited data collected in humans. This is important for the expected consequences of secondary retinal changes on vision restoration, although the general validity of laboratory findings for the human disease might be challenged by the substantial clinical differences among RP individuals.

In broad terms, the process of retinal degeneration comprises three stages: (1) primary photoreceptor loss; (2) secondary photoreceptor degeneration and (3) tissue remodeling. The latter consists of a gradual morphological, functional, and molecular reprogramming of all retinal components, comprising neurons, glial cells and blood vessels (Jones et al., 2005).

As in the case of any other part of the CNS, the retina of mammals is not capable of regenerating and therefore remodeling cannot be considered as an attempt of producing new cells or to re-grow their processes. At least, not a successful attempt. Rather, some of the events observed in remodeling are partially explainable by synaptic deafferentation of retinal cells deprived of photoreceptor input; some others are caused by local inflammatory reactions by resident and infiltrating microglia/macrophages; further rearrangements are due to the enlargement of macroglia to seal the space left vacant by dying cells. Remodeling is therefore mostly regressive and culminates with death of further cell types, some being more vulnerable than others. In RP, blood vessels also undergo a process of progressive atrophy, opposite to the vasculogenesis found in many retinal degenerations including diabetic retinopathy (also characterized by loss of retinal neurons) (Kastelan et al., 2013). The evidence of ischemic cytotoxicity is instead limited.

Altogether, remodeling in RP is both regressive and progressive. The kinetics differ greatly with the genetic mutation underlying the disease, which in turn influences the severity of the phenotypic advancement. But the end stage is inevitably characterized by cell death and extensive gliosis, with total disorganization of the regularly layered retinal architecture.

## A Quick Survey of Remodeling: Photoreceptors and Glia

In typical RP, rod photoreceptor death precedes cone photoreceptor loss. In some forms of retinal degeneration, cone photoreceptors die first, while in others cones can survive late into the disease process. No matter which one is the type of photoreceptor degenerating first, inner retinal rearrangements accompany and follow both the death of rods and cones. Surviving cones themselves remodel, typically forming cellular clusters showing sprouting of telodendria (John et al., 2000; Lin et al., 2009). Ectopic synapses (i.e., between residual cones and rod bipolar cells) might form. Glial mobilization in the outer retina is readily detectable concomitantly to the disappearance of photoreceptors: resident microglia is activated and cells move to the superficial layers to engulf and clear dying photoreceptors. Muller cells become hyperactive and hypertrophic, ultimately forming a gliotic scar that obliterates the normally present subretinal space (Cuenca et al., 2014). This poses potential challenges for obtaining surgical access to the outer retina (i.e., to deliver viral vectors) and to achieve electrical stimulation with electronic prostheses.

## Second Order Neurons

Studies on animal models of RP have shown that the earliest morphological signs of (extra-photoreceptor) remodeling appear in rod bipolar cells, directly connected to rods, the cells which normally die first. Typically, rod bipolars show major dendrite reduction, retraction and mislocation, and displacement of cell bodies, plus decreased expression of mGluR6 receptors and iGluR glutamate-activated currents (Strettoi and Pignatelli, 2000; Strettoi et al., 2003b; Gargini et al., 2007). This process is reminiscent of transynaptic atrophy and might be accompanied by new synaptic connections and temporary neuronal networks. Activation of the retinoic acid pathways and a phenotypical switch of ON-bipolar cells to OFF-bipolar cells have been described (Marc et al., 2007).

A remodeling sequence has emerged from morphological and functional studies on rodent models: rod bipolars remodel before cone bipolars, but ON cone bipolars rearrange before OFF cells, both anatomically and functionally (Puthussery et al., 2009). Evidently, the ON pathway, characterized by the expression of mGluR6 on bipolar cell dendrites, is more susceptible to the effects of photoreceptor death. Obviously, these changes are highly significant for the outcome of any gene therapy focusing on bipolar cells, for instance if mGluR6 is chosen as a promoter (Cronin et al., 2014).

Although less studied, horizontal cells are known to rearrange, similarly to other retinal neuron. Remarkably, their axonal arbors, postsynaptic to rods, undergo structural modifications (i.e., profuse sprouting in certain models and major regression in others) before their cell bodies and dendrites, which receive connections from cones (Strettoi et al., 2003a,b). Again, the effects observed in horizontal cells upon photoreceptor loss resemble anterograde transynaptic degeneration. In late stages, they also die secondarily.

## Amacrine and Ganglion Cells

Reactions of amacrine cells to the primary loss of photoreceptors have been studied in various animal models of RP (Marc et al., 2007). Similarly to cone bipolar cells, they remain relatively stable initially but might undergo changes in their chemical signature and engage in network of new, aberrant synaptic connections. These might contribute to oscillatory signals recorded from degenerating retinas and constituting a hallmark of animal models of RP (Stasheff et al., 2011). Yet, amacrine cells survive better than bipolar cells in the long run, and retain iGluR-mediated responsivity.

Ganglion cell modifications in RP have been described in detail, although mostly using phosphodiesterase mutants. Apparently, the only retinofugal neurons modify minimally upon photoreceptor loss, at least from the point of view of cell survival and dendritic maintenance, both considerably well preserved even in models tested at late stages of the disease (Mazzoni et al., 2008; Damiani et al., 2012; O’Brien et al., 2014). However, these neurons show peculiar functional abnormalities in the form of a paroxysmal electrical activity (Stasheff, 2008; Stasheff et al., 2011) that originates presynaptically (Margolis et al., 2008, 2014; Menzler and Zeck, 2011) and that might affect the outcome of the implant of restorative electronic prostheses.

Ganglion cells, perhaps the most stable retinal cells during retinal degeneration, and the last avenue for vision restoration based on a retinal approach, might represent a suitable target for optogenetic therapy in humans and clinical trials exploiting this possibility are approaching (Sahel and Roska, 2013).

## Perspectives

Given that remodeling does occur (in the retina, as in any other area of the diseased or injured CNS), several of the issues related to this complex biological process would benefit from further investigation. Indeed, even cells which ultimately remodel extensively, do show phases of remarkable preservation, which constitute possible therapeutic windows.

The molecular triggers of remodeling are largely unknown and the consequences of remodeling on therapeutic options postulated but not proven experimentally.

The search for molecular signals initiating remodeling should explore the robust glial reaction triggered by the primary death of rods in RP. This local, inflammatory process leads to major changes in gene expression and to the modulation of a high number of molecular species, known to create a hostile microenvironment (Yoshida et al., 2013). This could favor the secondary death of cones and accelerate inner retinal remodeling. Analysis of large cohorts of molecules (i.e., by RT-PCR, Next Generation Sequencing etc.) should be used to address this issue, also in view of the possibility to uncover new pathways for pharmacological therapies targeting inflammation (Guadagni et al., 2015).

Synaptic rewiring in the IPL (a process still poorly understood) should be studied systematically exploiting non-developmental animal models of RP, as in humans the disease typically manifests well beyond the phase of retinal development. Experiments employing prostheses stimulating ganglion cells are also necessary to learn whether and how the electronic implants are affected by the spontaneous firing of these cells.

Also, it would be important to learn whether and which ones of the main remodeling events can be reverted by therapy, as approaches that slow down photoreceptor degeneration translate into attenuation and delay of retinal remodeling as well (Piano et al., 2013). For instance, would optogenetic targeting of ganglion cells influence (and perhaps abolish) spontaneous firing?

Altogether, remodeling is an active field of retinal research, expected to continue growing in the near future.

## Author Contributions

This mini review was written as a general introduction to the articles contributed by different Authors to a special topic issue. The review contains original considerations elaborated thanks to the years spent doing retinal research in the field of inherited degenerations, within one of the laboratories in which retinal remodeling has been studied first.

## Conflict of Interest Statement

The author declares that the research was conducted in the absence of any commercial or financial relationships that could be construed as a potential conflict of interest.
